# Exploration of the recurrence in radiation brain necrosis after bevacizumab discontinuation

**DOI:** 10.18632/oncotarget.7768

**Published:** 2016-02-26

**Authors:** Hongqing Zhuang, Xiangkun Yuan, Joe Y. Chang, Yongchun Song, Junjie Wang, Zhiyong Yuan, Xiaoguang Wang, Ping Wang

**Affiliations:** ^1^ Department of Radiotherapy, Tianjin Medical University Cancer Institute and Hospital, National Clinical Research Center for Cancer, Tianjin Key Laboratory of Cancer Prevention and Therapy, Tianjin, China; ^2^ Department of Radiotherapy, Hebei Province Cangzhou Hospital of Integrated Traditional and Western Medicine (Cangzhou No. 2 Hospital), Hebei, China; ^3^ Department of Radiation Oncology, Division of Radiation Oncology, The University of Texas MD Anderson Cancer Center, Houston, Texas, United States of America; ^4^ Department of Radiotherapy, Peking University 3rd Hospital, Beijing, China

**Keywords:** bevacizumab, brain necrosis, recurrence

## Abstract

Objective: The aim of the paper was to investigate the recurrence and its causes of radiation brain necrosis following bevacizumab discontinuation. Methods: This study included 14 patients with radiation brain necrosis (confirmed through imaging) after stereotactic radiotherapy for a primary or metastatic brain tumor and who received bevacizumab treatment from June 2011 through December 2014. The patients received bevacizumab at 5 mg/kg, q3-4w, for at least 3 cycles. The T1 signal intensity from enhanced MRI images was used as the evaluation criteria for the brain necrosis treatment efficacy. Results: brain necrosis improved in 13 of the 14 cases (92.9%). However, during follow-up, 10 of the 13 responsive patients (76.9%) exhibited a recurrence in brain necrosis, and a multiple linear regression analysis shows that brain necrosis recurrence was related to the follow-up time after the initial bevacizumab treatment discontinuation. Conclusion: bevacizumab produced good short-term effects for radiation brain necrosis; however, most of the patients would recurrence after bevacizumab is discontinued. Thus, brain necrosis was irreversible.

## INTRODUCTION

Radiation brain necrosis is a common complication after brain tumor radiotherapy [1–3]. Many clinical studies have shown that bevacizumab produces good short-term effects on radiation brain necrosis [4–11]. However, few studies have reported on radiation brain necrosis progression following bevacizumab discontinuation, and the causes of such progression have not been studied. To provide a reference for clinical treatment, we statistically analyzed bevacizumab treatment efficacy in patients with radiation brain necrosis treated at the Tianjin Cancer Hospital and their progression after bevacizumab discontinuation.

## MATERIALS AND METHODS

### Clinical data

This study was approved and completed with oversight by the Ethics Committee of Tianjin Tumor Hospital. The study complied with all ethics regulations. All patients signed an informed consent form. The inclusion criteria were primary or metastatic brain lesions; a history of CyberKnife stereotactic radiotherapy for brain lesions; and radiation brain necrosis confirmed by imaging or pathology. We studied 14 patients with radiation brain necrosis after stereotactic radiotherapy for a primary or metastatic brain tumor and who received bevacizumab treatment from June 2011 and December 2014. The patients included 6 males and 8 females, 31-70 years old (median: 53 years old). None of the cases presented a primary brain tumor, but the 14 cases presented metastatic brain tumors (11 cases of brain metastasis from lung cancer, 1 case of brain metastasis from breast cancer, 1 case of brain metastasis from gastric cancer and 1 case of brain metastasis from lymphoma). Four patients had previously undergone whole-brain radiotherapy. The stereotactic radiotherapy was provided as follows:50-82% of the dose limit (median:75%),1,400-4,000cGy(median:2,600cGy), and bioequivalent dose of 5,980-9,880cGy(median:7,590cGy) provided in 1-5 treatments (median: 2 treatments). The patients received 3-10 bevacizumab treatment cycles (median:3cycles) (Table 1).

**Table 1:** Base line of patients

Characteristic	Value
Cases	14
Gender(cases/percent) male Female	6/43 8/57
Age(year) Range Media	31-70 56
Primary or metastases (cases/percent) Primary Metastases	0 14
Primary site (cases/percent) Lung Breast Lymphoma Gastric cancer	11/78.6 1/7.1 1/7.1 1/7.1
WBRT (cases/percent) Yes No	4/28.6 10/71.4
Dose(cGy) Range Media	1400-4000 2600
Dose line(%) Range Media	50-82 75
Fractions Range Media	1-5 2
BED (cGy) Range Media	5980-9880 7590
History of bevacizumab use Yes No	2 12
frequency of bevacizumab Range Media	3-10 3
Dose of bevacizumab	5mg/kg q3-4w
Follow time(months) Range Media	4.3-39.1 12.3

### Clinical diagnostic criteria for brain necrosis

A pathological diagnosis remains the gold standard for diagnosing radiation brain necrosis; however, many issues arise in clinical practice. First, with stereotactic radiotherapy, many brain tumors are close to the base of the skull or in important functional areas, which eliminates surgical resection as well as stereotactic biopsy and, thus, a potential pathological diagnosis. Second, few patients are willing to undergo a biopsy after stereotactic radiotherapy. Third, a stereotactic biopsy may not provide a full pathological picture of the tumor tissue. Moreover, it is difficult to ask patients with multiple intracranial metastases and who receive palliative treatment to undergo a craniotomy to confirm a diagnosis if brain necrosis is suspected. And in such patients, a craniotomy is inconsistent with the treatment goal of prolonging survival and improving quality of life. Hence, although a post-operative pathological diagnosis is the gold standard for diagnosing radiation brain necrosis, it is difficult to implement in clinical practice. Thus, a comprehensive imaging modality is the most practical and common diagnostic method for radiation brain necrosis in clinical practice [12]. In this study, the patients were diagnosed based on a comprehensive medical history, symptoms, signs, MRI, spectroscopy and positron emission tomography-computed tomography (PET-CT) [13–20]. MRI or spectroscopy was performed first, and then, PET-CT was performed as needed for ambivalent diagnoses. Moreover, the tests were repeated to monitor for changes. Most brain necrosis cases exhibited irregular low T1WI and irregular high T2WIsignals in the MRI scans. Brain necrosis manifested as low T1WIand high T2WI signals. For the enhanced scans, the lesion center showed irregular enhancement without enhanced nodules. Further, the T1 and T2 signals indicated a large edema area surrounding the lesion with no enhancement. MRS showed low Cho, Cr and NAA levels as well as markedly lower NAA/Cho and NAA/Cr ratios in the radiation brain necrosis lesions. PET showed a lower metabolic rate in the radiation brain necrosis lesions than in the normal brain tissue and a lower glucose uptake rate in the corresponding lesion area. An imaging-based brain necrosis diagnosis must be confirmed by radiologists.

### Bevacizumab treatment

Because the decisive factor of anti-vascular therapy was not the concentration but duration of drug action. So, all the patients received bevacizumab at 5 mg/kg, q3-4w/cycle. Before the bevacizumab treatment, patients received an anti-allergy treatment with diphenhydramine and steroids. The patients received the initial bevacizumab dose over 90 minutes and the subsequent doses over 60 minutes. During administration, the patients were monitored using ECG to closely observe drug responses. Once brain necrosis was diagnosed, the patients received at least 3 cycles of bevacizumab treatment during the first course. If the treatment was effective for brain necrosis after three cycles, bevacizumab was or was not discontinued. For brain necrosis progression following bevacizumab discontinuation, whether to re-treat with bevacizumab (same dose and administration) was determined based on the patient's conditions and wishes.

### Evaluation criteria before and after treatment as well as for brain necrosis recurrence

A routine brain MRI scan was performed one month after 3 cycles of bevacizumab treatment, which was followed by scans every 2-3months during the next year and then as needed based on the patient's condition, but the interval did not exceed six months. Moreover, an MRI scan was promptly performed where the patient presented intracranial symptoms.T1-weighted signal changes in the brain necrosis area of an enhanced MRI scan were used to evaluate the treatment results and brain necrosis recurrence. Three areas from the enhanced brain necrosis area were used to measure the signal intensity and calculate the mean value, which was then compared with the white matter signal intensity for the same MRI scan to eliminate the effects from the enhancement. The adjusted value was then used to measure the signal intensity changes for the brain necrosis area before and after treatment [21]. 5. Statistical analysis

SPSS17.0 software was used for statistical analyses. *P* < 0.05 was considered statistically significant. A t test was performed to analyze the brain MRIsignal intensity changes before and after the first course of bevacizumab treatment. A rank sum test was performed to analyze difference factors in patients with and without brain necrosis recurrence. Logistic regression analysis was used to analyze the factors related to brain necrosis recurrence after bevacizumab treatment.

## RESULTS

### Bevacizumab treatment results

The T1-weighted signal intensity of the brain necrosis area from an enhanced MRI scan was analyzed before and after bevacizumab treatment, and brain necrosis improved in 13 of the 14patients(92.86%)after treatment (Table 2), with a significant change in brain necrosis before and after treatment(*t* = 4.507, *P* = 0.001).

**Table 2:** The effect of bevacizumab and the characters of patients' recurrence after bevacizumab discontinuation

Characteristic	Value
Effective of the first course bevacizumab(Cases/percent) Yes No	13/92.9 1/7.1
The maintain time of remission after the first course bevacizumab (from the first cycle of bevacizumab, months)	
Range Media	2-11.1 7.1
Recurrence in the effective patients (Cases/percent)^a^ Yes No	10/76.9 3/13.1
Re-treatment with bevacizumab after recurrence (Cases/percent) Yes No	5/50 5/50
The cycles of re-treatment with bevacizumab Range Media	1-7 2
The effect of re-treatment with bevacizumab (Cases/percent) Yes No	3/60 2/40
maintain time of re-treatment in the effective patients (months) Range Media	1.9-4.0 2.5
Follow up time after the discontinuation of first course (months)	
Range Media	1.3-38.0 10.0
Total follow up time(months) Range Media	4.3-39.1 12.3

a The increase or decrease of enhanced MRI signal was used to evaluate the remission and relapse of radiation necrosis.

### Data for patients with radiation brain necrosis progression after bevacizumab discontinuation

For the 13 patients who responded to bevacizumab treatment, the median follow-up time after bevacizumab was discontinued following the first-course treatment was10.0 months (1.2-38.0 months),and 10patients (76.9%) recurrence. The 3 patients without recurrence presented advanced brain metastases; 2 patients died shortly after bevacizumab was discontinued (2.3months and 1.2months, respectively),and 1 patient only followed up for a short period of time after bevacizumab was discontinued(2.5 months). Among the11 patients who recurrenced,5 patients received re-treatment with bevacizumab, and 3 patients responded to re-treatment (Table 2).

### A univariate analysis of the causes for radiation brain necrosis progression following bevacizumab discontinuation

We performed a comparative analysis of the patients who responded to the first course of bevacizumab treatment and considered factors such as the biologically effective dose (BED) of stereotactic radiotherapy, number of cycles during the first course of bevacizumab treatment, brain necrosis volume before bevacizumab treatment, brain necrosis residual volume after the first course of bevacizumab treatment, and follow-up time after bevacizumab was discontinued following the first course of treatment. Based on this analysis, between patients with brain necrosis recurrence and without, we only observed a significant difference for follow-up time after bevacizumab was discontinued following the first course of treatment (Table 3).A logistic regression analysis of the relationship between these factors and brain necrosis recurrence showed that the follow-up time after bevacizumab was discontinued following the first course of treatment was the only factor related to brain necrosis recurrence (Table 4). These results suggest that brain necrosis is irreversible and that in time, all patients will present brain necrosis recurrence.

**Table 3:** The rank sum test results of related factors in recurrence and no recurrence patients

	*P* value
BED	0.88
The medication cycles of first course with bevacizumab	0.47
Volume of necrosis before bevacizumab treatment	1.00
Residual necrosis after the first course bevacizumab treatment	0.16
Follow up time after the first course bevacizumab treatment	0.007

**Table 4:** The logistics regression analysis of the factors associated with brain necrosis recurrence

	*P* value
Gender	0.84
Age	0.87
Bevacizumab treatment history	0.40
BED	0.73
Primary tumor location	0.79
The medication cycles of first course with bevacizumab	0.46
Volume of necrosis before bevacizumab treatment	0.92
Residual necrosis after the first course bevacizumab treatment	0.38
Follow up time after the first course bevacizumab treatment	0.032

### Imaging data for the representative cases of radiation brain necrosis progression following bevacizumab discontinuation

**Figure 1:**
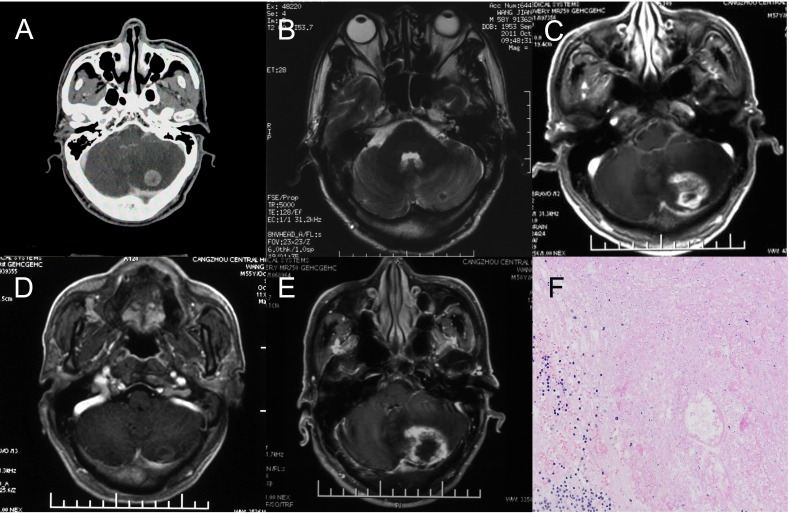
One case of brain necrosis recurrence after bevacizumab discontinuation **A.** The metastasis lesion before Cyberknife treatment. **B.** 3 months after Cyberknife treatment. The lesion significantly reduced, almost disappeared. **C.** 14 months after Cyberknife treatment. The brain necrosis occurred and the bevacizumab was used. **D.** the patient received bevacizumab at 5 mg/kg, q3w× 3cycles.A brain MRI scan showed significant improvement in the necrosis and then the bevacizumab discontinued (nearly disappeared). **E.** 5 Months after the bevacizumab discontinuation, MRI scans showed a large area with enhancement and edema at the original treatment site. The diagnosis by radiologists was cerebral necrosis relapse. **F.** A Local excision was treated and the pathological examination confirmed radiation brain necrosis.

Data for 1 patient was used to further develop an in-depth understanding of radiation brain necrosis recurrence following bevacizumab discontinuation. A 58-year-old male patient visited the hospital due to cerebellar metastasis of lung cancer and underwent CyberKnife stereotactic radiotherapy (dose: 23 Gy/1f) for brain metastasis in June 2011.A follow-up visit in October 2011 showed a significantly smaller cerebellar lesion. However, a follow-up visit in August 2012showed significant enhancement at the original treatment site with a large edema area in the surrounding tissue. The patient was diagnosed with radiation brain necrosis based on imaging. In 2012, the patient received bevacizumab at 5 mg/kg, q3w× 3cycles.A brain MRI scan performed in October 2012 showed significant improvement in the radiation brain necrosis (nearly disappeared). However, a brain MRI scan performed in April 2013 showed a large area with enhancement and edema at the original treatment site, and a postoperative pathological examination confirmed radiation brain necrosis (Figure 1).

## DISCUSSION

This study shows that bevacizumab is effective for treating radiation brain necrosis; however, most patients exhibit recurrence and progression after bevacizumab is discontinued, and radiation brain necrosis is essentially irreversible.

The mechanisms underlying effective bevacizumab treatment of radiation brain necrosis involve underlying radiation brain necrosis development and bevacizumab anti-angiogenesis effects [22–23]. The vascular injury mechanism plays an important role in radiation brain necrosis development. Radiation destroy blood vessels through cellulose-like changes in vascular endothelial cells, yielding tissue hypoxia as well as necrosis and an ensuing release of high levels of vascular cytokines (e.g., vascular endothelial growth factor [VEGF]). Such changes gradually lead to blood-brain barrier disorder and brain edema, thereby affecting certain neurological functions. Hence, bevacizumab is targeted to preventing and treating radiation brain necrosis by blocking VEGF release into target vessels and reducing vascular permeability, the quantity of inflammatory cytokines in plasma and plasma flow and blood-brain barrier damage and brain edema.

Currently, research on brain necrosis progression mechanisms after discontinuing bevacizumab is inadequate. Certain case reports indicate that during brain necrosis treatment, bevacizumab may further reduce the blood supply to the brain necrosis area through anti-angiogenic mechanisms, which causes localized ischemia and hypoxia, resulting in brain necrosis recurrence [24]. However, bevacizumab is an anti-angiogenic drug recognized for its role in pruning vessels and promoting blood supply, which is inconsistent with the above inference [25]. We believe that several mechanisms are involved in radiation brain necrosis recurrence. First, radiation brain necrosis is caused by several mechanisms, and vascular injury is only one such mechanism. Both glial cell damage and immune reactions are potentially important mechanisms; therefore, bevacizumab can only target one mechanism. Second, the pathological changes due to necrosis are irreversible. Bevacizumab does not treat the root cause of brain necrosis; instead, it relies on anti-angiogenic effects to reduce vascular permeability and edema in the brain necrosis area. Once withdrawn, bevacizumab's effects gradually decrease and eventually disappear. However, the pathological basis underlying brain necrosis remains intact, thereby re-initiating reactions that stimulate new blood vessel formation, which is demonstrated through the reappearance of vascular proliferation around the necrosis as well as brain necrosis recurrence and progression in imaging experiments [26]. Among many previous studies, only a few have reported several recurrence cases [4–10]. Our literature review shows that previous researchers may have missed progression in many cases reported as “without progression” due to individual differences in bevacizumab sensitivity, short follow-up times after bevacizumab is discontinued, or death shortly after bevacizumab is discontinued.

Compared to previous reports, most studies [4–10] only reported the efficacy of bevacizumab in the treatment of radiation-induced cerebral necrosis, while there were fewer studies on the recurrence and progression of radiation-induced cerebral necrosis after treatment. No studies have yet explored the reasons for recurrence and progression. This study preliminarily investigated the efficacy of bevacizumab in the treatment of radiation-induced cerebral necrosis and the progression conditions and reasons after drug withdrawal, which provided new references for clinical practices and proposed new topics for the treatment of radiation-induced cerebral necrosis with bevacizumab.

Overall, through the analysis of bevacizumab efficacy in radiation-induced cerebral necrosis and disease progression after drug withdrawal, this study provides new references for the clinical practice of treatment of radiation-induced cerebral necrosis with bevacizumab. Although a large number of case reports have been difficult to establish in the treatment of radiation-induced cerebral necrosis with bevacizumab, we believe that, provided more results are published in study reports, there will be a better understanding of the treatment of radiation-induced cerebral necrosis with bevacizumab.
